# Sources and Characteristics of Particulate Matter in Subway Tunnels in Seoul, Korea

**DOI:** 10.3390/ijerph15112534

**Published:** 2018-11-12

**Authors:** Yongil Lee, Young-Chul Lee, Taesung Kim, Jin Seok Choi, Duckshin Park

**Affiliations:** 1 Korea Railroad Research Institute (KRRI), 176 Cheoldobakmulkwan-ro, Uiwang-si 16105, Korea; freego83@krri.re.kr; 2 Mechanical Engineering, Sungkyunkwan University, 2066 Seobu-ro, Suwon-si 16419, Korea; tkim@skku.edu; 3 Department of BioNano Technology, Gachon University, 1342 seongnamdae-ro, Seongnam-si 13120, Korea; dreamdbs@gachon.ac.kr; 4 Analysis Center for Research Advancement, Korea Advanced Institute of Science and Technology (KAIST), 291 Daehak-ro, Yuseong-gu, Daejeon-si 34141, Korea; ffband@kaist.ac.kr

**Keywords:** characteristics, particulate matter, source identification, subway tunnel, air quality

## Abstract

Hazards related to particulate matter (PM) in subway systems necessitate improvement of the air quality. As a first step toward establishing a management strategy, we assessed the physicochemical characteristics of PM in a subway system in Seoul, South Korea. The mean mass of PM_10_ and PM_2.5_ concentrations (*n* = 13) were 213.7 ± 50.4 and 78.4 ± 8.8 µg/m^3^, with 86.0% and 85.9% of mass concentration. Chemical analysis using a thermal–optical elemental/organic carbon (EC–OC) analyzer, ion chromatography (IC), and inductively coupled plasma (ICP) spectroscopy indicated that the chemical components in the subway tunnel comprised 86.0% and 85.9% mass concentration of PM_10_ and PM_2.5_. Fe was the most abundant element in subway tunnels, accounting for higher proportions of PM, and was detected in PM with diameters >94 nm. Fe was present mostly as iron oxides, which were emitted from the wheel–rail–brake and pantograph–catenary wire interfaces. Copper particles were 96–150 nm in diameter and were likely emitted via catenary wire arc discharges. Furthermore, X-ray diffraction analysis (XRD) showed that the PM in subway tunnels was composed of calcium carbonate (CaCO_3_), quartz (SiO_2_), and iron oxides (hematite (*α*-Fe_2_O_3_) and maghemite-C (*γ*-Fe_2_O_3_)). Transmission electron microscopy images revealed that the PM in subway tunnels existed as agglomerates of iron oxide particle clusters a few nanometers in diameter, which were presumably generated at the aforementioned interfaces and subsequently attached onto other PM, enabling the growth of aggregates. Our results can help inform the management of PM sources from subway operation.

## 1. Introduction

Subway systems relieve traffic congestion in metropolitan areas as an environmentally friendly means of transportation [1,2,3,4,5]; however, human exposure to air pollutants in subway systems is a concern [6]. Particulate matter (PM) exerts detrimental effects on health. In 2013, the specialized cancer agency of the World Health Organization (WHO), the International Agency for Research on Cancer (IARC), announced that it classified outdoor air pollution as carcinogenic to humans (Group 1) [7]. For instance, a 10 µg/m^3^ decrease in PM_10_ concentration resulted in an 8.36% drop in the all-cause mortality rate in Beijing, China [8], and the mortality risk estimated in a Dutch cohort study for PM_2.5_ was 6% per 10 µg/m^3^ for natural-cause mortality [9].

Moreover, when PM is inhaled by the respiratory tract, smaller-diameter PM can penetrate the lung (alveolus) [10]. PM in subway systems is more harmful than other sources, e.g., wood combustion, traffic (vehicles), and roadways, due to redox active iron on the surface of the subway particles [11,12]. Specifically, in the literature [13], magnetite (Fe_3_O_4_) was observed in the human brain.

The Seoul Metro Company installed platform screen doors (PSDs) at all stations, a feature which is also being implemented in other nations. PSDs reduce the risk of accidents and increase the operation efficiency of heating, ventilation, and air-conditioning systems [14,15]. As the installation of PSDs results in the isolation of the platform from the tunnel, the PM level on the platform is significantly reduced [15]; however, the accumulation of PM in the subway tunnel worsens the air quality therein [16].

The PM in subway tunnels originates from operation of the subway, ventilation, and re-suspension of settled PM [2,16,17,18,19]. Many studies reported high levels of PM in subway tunnels and determined its chemical composition [6]. Metals are the major constituents of PM in subway tunnels [16,20,21,22,23], principally Fe [16,22,23,24]. According to previous field studies, wear PM with peak diameters of 100, 350, and 300–700 nm is emitted during mechanical braking [3]. In addition, wear PM with peak diameters of 6.98 and 165.5 nm is emitted from the wheel–rail interface during electrical braking [2]. This PM in subway tunnels could penetrate the subway cabin via heating, ventilation, and air-conditioning (HVAC) systems and outflow via the ventilation system in the tunnel into the city.

The secondary pollutants (SO_4_^2−^, NO_3_^−^, and NH_4_^+^) in PM are photochemically converted from gases and comprise 2.4% of PM_10_ and 3.7% of PM_2.5_. In particular, SO_4_^2−^ is generated by secondary chemical reactions in the atmosphere [25,26], whereas NO_3_^−^ is generated by combustion of fossil fuels [27]. When the subway service is inactive, a diesel motor car is operated to maintain the tunnel, and NO_3_^−^ and SO_4_^2−^ are transported from the outdoor air through the ventilation system [2,16,28,29,30,31,32].

Information on the chemical properties and sources of PM in subway tunnels is needed to facilitate an effective management strategy and thereby improve the air quality. In this study, we characterized the PM in subway tunnels beneath Seoul, Korea.

## 2. Materials and Methods

### 2.1. Study Area

**Figure 1:**
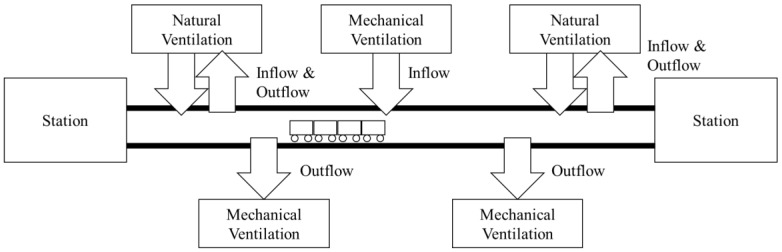
The natural and mechanical ventilation systems in line 4.

In 2016, the Seoul metropolitan subway transported 7.4 million passengers daily. The Seoul Metro Company operates subway lines 1 to 8, and other companies operate line 9, the airport line, and the Bundang line. Line 4 is connected to the Danggogeo station in Seoul and the Oido station in Ansan, Geyonggi-do, consisting of an overground and underground section. The underground section runs from Beomgye to Chongshin University station and from Sinyongsan to Ssangmun station. The subway tunnels in line 4 consist of natural and mechanical ventilation systems, as shown Figure 1. When the mechanical ventilation system is not in operation, it can use natural ventilation.

**Figure 2:**
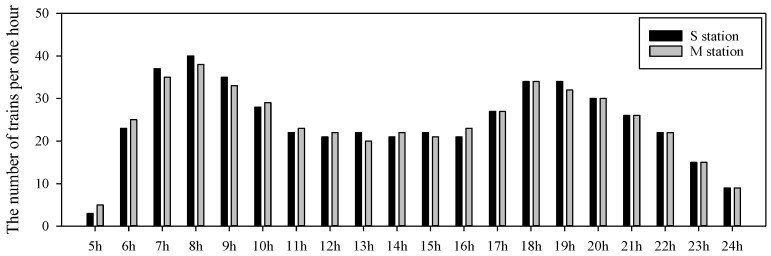
Number of trains at the M station and S station on weekdays.

**Figure 3:**
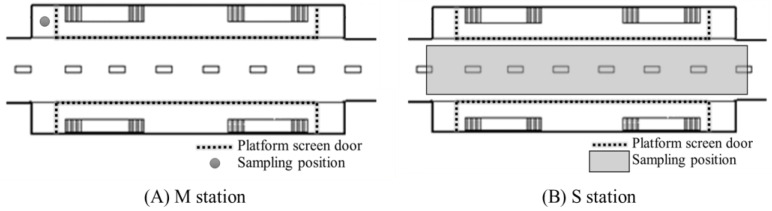
Positions of the sampling sites at the M (**A**) and S (**B**) stations.

We analyzed the PM in a subway tunnel at the M station (60,000 passengers/day) and S station (27,000 passengers/day), which handle 490 trains per day with eight carriages (Figure 2). Figure 3 shows sampling positions on the platform equipped with PSDs.

### 2.2. Collection and Analysis of Samples

Table 1 lists the measurement devices and sampling periods. To analyze the inorganic, ionic, and carbonaceous components of PM, samples were collected in the subway tunnels at the M station from 15 May to 9 June 2017. PM samples were collected on quartz filters (QMA filter, 47 mm in diameter; Pall Corporation, New York, NY, USA) using a mini-volume air sampler (MiniVol TAS, 5 L/min; Airmetrics, Eugene, OR, USA) and Zefluor filters (polytetrafluoroethylene membrane filter, 47 mm in diameter; Pall Corporation, New York, NY, USA) using a low-volume air sampler (PMS-104, 16.7 L/min; APM, Bucheon, Korea). For the X-ray diffraction (XRD) analysis, samples were collected from the bottom of the subway tunnels at the S station. The quartz and Zefluor filters were changed daily at 5:00 p.m. Lastly, for the transmission electron microscopy analysis, samples were collected in the subway tunnels on 18 April 2017.

To analyze the chemical composition and morphology of PM, an aluminum foil filter (CFG-225, 25 mm in diameter; Dekati, Kangasala, Finland) and electrical low-pressure impactor (ELPI, 10 L/min; Dekati, Kangasala, Finland) were used to collect PM samples from 15 May to 17 May 2017. The size distribution of PM was also stored in high-resolution ELPI+ mode (HR-ELPI+). The floor dust in the subway tunnels was collected and passed through a 1-mm (18 mesh) filter to identify crystalline material.

Zefluor filters were weighed before and after sampling using an analytical balance (sensitivity: 0.001 mg) after being equilibrated at constant temperature and humidity for three days in an electronic desiccator. Quartz filters were heated to 850 °C for >2 h to remove organic matter before sampling.

After PM sampling, the Zefluor filters were cut in half using ceramic scissors. Each half was preprocessed for analysis of the inorganic and ionic components. Preprocessing for the inorganic component analysis was done using inductively coupled plasma (ICP) spectroscopy, and complied with the preprocessing standard of the Clean Water Act of the United States. The sample was placed in a perfluoroalkoxy polymer resin liner and mixed with 7 mL of 61% nitric acid and 3 mL of 35% hydrochloric acid. The mixture was then subjected to a pressure of 150 psi for 10 min to extract inorganic components. The extracted solution was filtered (number 5B filter paper, 110 mm; Advance MFS), diluted with 50 mL of ultrapure water, and stored at 4 °C until required. The Fe, Ni, Ba, Pb, V, Cr, Cu, Zn, Mn, and Al contents were analyzed using ICP atomic emission spectrometry (ICP-AES; ICPE-9000, Shimadzu, Japan).

For analysis of the water-soluble ion content, the filter paper was soaked in 20 mL of ultrapure water, and ions were extracted using an ultrasonic extractor. To prevent clogging of the ion chromatography (IC) column, the extracted solution was filtered through a 0.45-µm SCA cellulose acetate syringe filter (CHMLAB Group, Barcelona, Spain); the filtered solution was stored in a 60-mL narrow-mouth bottle (Nalgene, Waltham, MA, USA) at 4 °C. The water-soluble ion contents of the extracts were analyzed by IC (861 Advanced Compact IC; Metrohm, Herisau, Switherland). The NO_3_^−^, SO_4_^2−^, and Cl^−^ anions were analyzed using MetroSep A Supp 5 and MetroSep RP 2 Guard columns, and the NH_4_^+^, Na^+^, K^+^, Mg^2+^, and Ca^2+^ cations were analyzed using MetroSep C 4 and RP 2 guard columns.

A thermal–optical elemental/organic carbon (EC–OC) analyzer (Sunset Laboratory Inc., Parsippany, NJ, USA) was used to determine the organic carbon (OC) and elemental carbon (EC) contents following a thermal/optical transmittance (TOT) protocol [33]. The sample was heated to 900 °C in five steps in an He atmosphere to volatilize the OC and EC (Table 2). To evolve the EC and pyrolyzed OC, which were removed in the second part of the analysis, the sample was first cooled to 550 °C and heated in 2% O_2_/98% He to 910 °C in five steps. The analyzer utilizes laser transmission to correct for OC charring. The EC was determined as the C evolved after filter transmittance returned to the initial value.

PM morphology was characterized by scanning electron microscopy (SEM; S-4700; Hitachi, Tokyo, Japan) and transmission electron microscopy (TEM; Talos F200X; FEI, Hillsboro, OR, USA). Energy-dispersive spectroscopy (EDX) was used to analyze the chemical composition of PM. PM samples on grids were sputter-coated with Pt prior to SEM. For TEM and EDX analysis, the filters were placed in tubes, mixed with 20 mL of deionized water, dispersed using an ultrasonic extractor, and transferred to a TEM grid (CF200 Cu-C film, 200 mesh; EMS, Hatfield, PA, USA) using a pipette.

For the XRD analysis (SmartLab; Rigaku, Austin, TX, USA), a 1-mm sieve was used to filter dust collected from the floor of subway tunnels.

Quality assurance of the ICP-AES, IC, and C analyses was performed using standard solutions. Accuracy was checked by calculating the relative error, and precision was checked by calculating the relative standard deviations (RSDs) and coefficients of variation of triplicate determinations. For the C analysis, the RSDs were calculated by analyzing the same sample in triplicate, as no standards that enable OC and EC to be distinguished are available.

### 2.3. Estimated Carbonate by Ion Balance

Atmospheric PM consists of cations (Na^+^, NH_4_^+^, K^+^, Mg^2+^, and Ca^2+^), anions (CO_3_^2−^, Cl^−^, NO_3_^−^, and SO_4_^2−^), and trace amounts of organic acids and metal cations. We used the ion balance to evaluate the acid–base balance. We were unable to evaluate the carbonate contents of PM by IC. Therefore, we estimated the carbonate contents of PM as the difference between the contents of all other anions and cations, as shown in Equation (1) [34].

CO_3_^2−^ = (Na^+^ + NH_4_^+^ + K^+^ + Mg^2+^ + Ca^2+^) − (Cl^−^ + NO_3_^−^ + SO_4_^2−^). (1)

The normal concentration of carbonate estimated by ion balance was converted into mass concentration, and the carbonate carbon (CC) concentrations were subtracted from the organic carbon (OC) concentrations to avoid overlap with the carbonate estimated from the ion balance in Equation (1) [29].

## 3. Results and Discussion

### 3.1. PM Mass

In Seoul, air-quality monitoring stations (AQMS) are operated at 25 points, and their data are open-access. Thus, for the comparison of mass concentration outdoors and in subway tunnels, we collected PM_10_ and PM_2.5_ from the AQMS nearest to the M station during the measurement period.

**Figure 4:**
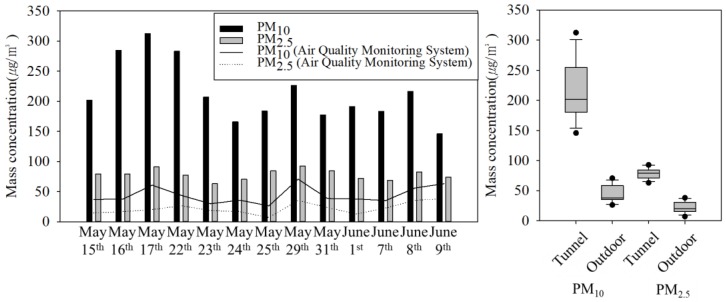
Particulate matter (PM) mass concentration in the subway tunnel at the M station.

The mean masses of PM_10_ and PM_2.5_ concentrations (*n* = 13) were 213.7 ± 50.4 and 78.4 ± 8.8 µg/m^3^ in the subway tunnels and 44.0 ± 13.9 and 22.2 ± 9.5 µg/m^3^ in outdoor air at the AQMS (*n* = 13), respectively (Figure 4). The mass concentrations of PM_10_ and PM_2.5_ in the subway tunnel were 4.8- and 2.5-fold greater than their counterparts in outdoor air. The PM levels in the subway tunnels exceeded the WHO air-quality guidelines (PM_10_, 24 h, 50 µg/m^3^; PM_2.5_, 24 h, 25 µg/m^3^) [35]. In the subway tunnels, the PM_10_ mass concentration was 2.5-fold greater than that of PM_2.5_. As shown in Figure 4, the concentration in the subway tunnel increased as the concentration outdoors increased. For comparisons between the tunnel and outdoor concentrations of PM, an independent *t*-test was used according to the data distribution with the SPSS 24 software (IBM Corporation, New York, NY, USA). The independent *t*-test resulted in *t*-values of 15.7 and 11.7 for PM_10_ and PM_2.5_, respectively, and *p*-values lower than 0.001 for both, indicating a highly significant difference between the subway tunnel and outdoors.

PM_10_ originates from the wheel–rail–brake pad interface due to mechanical wear caused by the structural characteristics of the subway tunnels [5,29,36]. According to Park et al. [16], 67.7% of PM_10_ originates from the wheel–rail–brake and catenary interfaces in subway tunnels. In addition, the PM concentration is influenced by train wind, the frequency of train operation, and tunnel cleaning [19]. The daily PM_10_ concentrations in the subway tunnel were quite different. As the measurement was only conducted on weekdays, it could be considered that the PM_10_ in the subway tunnel was diluted due to the operation of mechanical ventilation [37]. The PM_10_ concentrations in subway tunnels were reported to be 232–338 µg/m^3^ in Seoul and 51–470 µg/m^3^ in other countries [16,21,38,39,40]. For comparison, the mean PM_10_ mass concentration at the M station in 2014 was 184 µg/m^3^ [29], which is slightly lower than the finding in this study. The mechanical ventilation near the M station was improved in January 2017, and it could be considered to be the difference in cleanliness in the subway tunnels.

**Figure 5:**
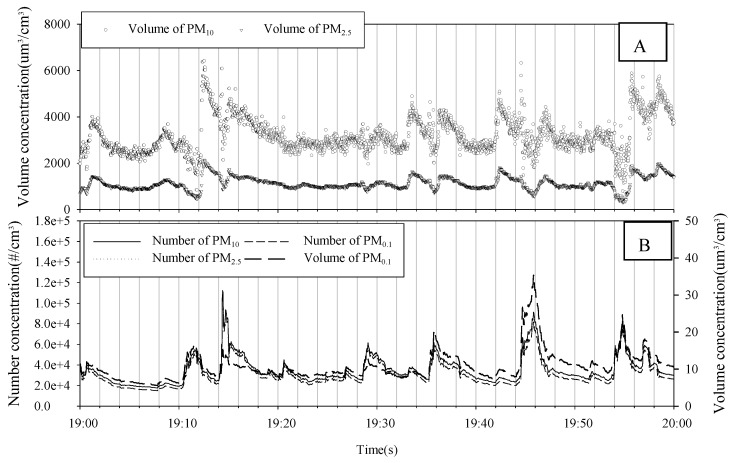
The number and volume of PM in the subway tunnel at the M station between 7:00 and 8:00 p.m. on 15 May 2017.

**Figure 6:**
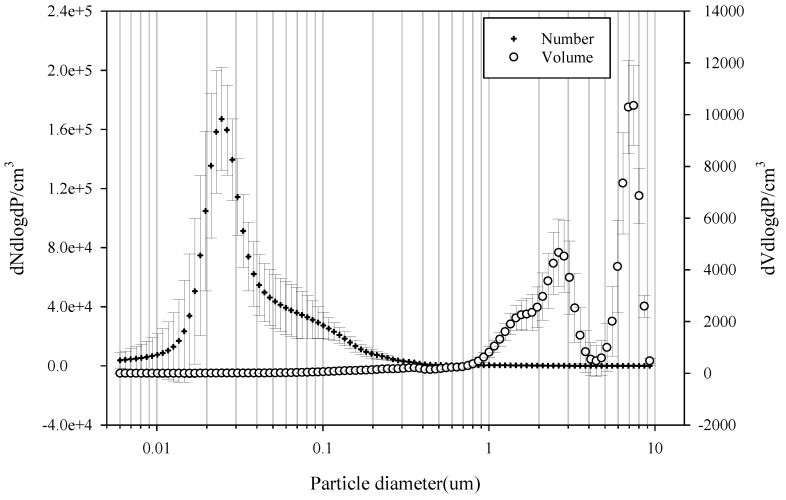
Size distribution of PM in the subway tunnel at the M station between 7:00 and 8:00 p.m. on 15 May 2017.

Figure 5 and Figure 6 show the number, volume, and size distribution of PM in the subway tunnel at the M station between 7:00 and 8:00 p.m. on 15 May 2017. In the M station, a total of 36 trains stopped during that time. The number and volume of PM varied in a constant pattern. It is thought that the particles were generated from the subway and resuspended by the piston effect in the subway tunnels [19]. The volume in Figure 5A shows a similar pattern, as does the volume and number of PM in Figure 5B. However, the pattern of Figure 5A compared to Figure 5B is different. This is due to the size distribution. The size distribution of the volume shows a bimodal shape with peaks at around 2–3 µm and 7–8 µm, while the number distribution shows a unimodal shape with a peak at around 0.02–0.03 µm.

### 3.2. Chemical Composition of PM

#### 3.2.1. Carbonaceous Compounds

**Figure 7:**
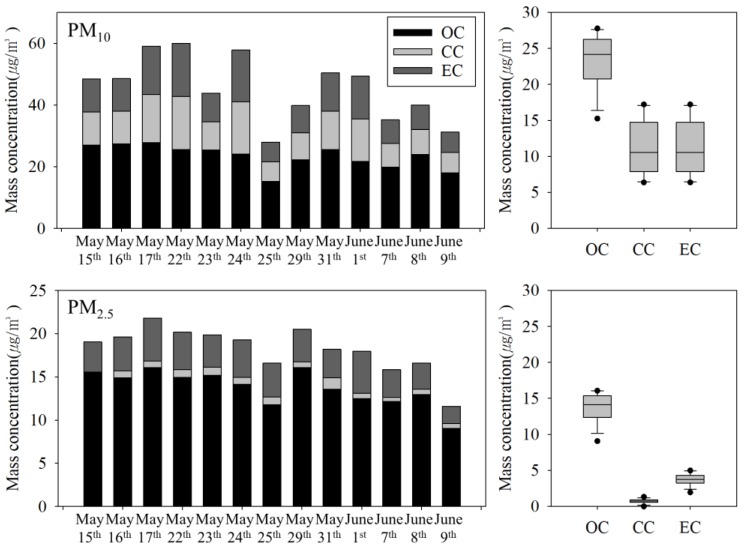
Mass concentrations of the carbonaceous components of PM in the subway tunnel at the M station (*n* = 13). OC—organic carbon; CC—carbonate carbon; EC—elemental carbon.

**Figure 8:**
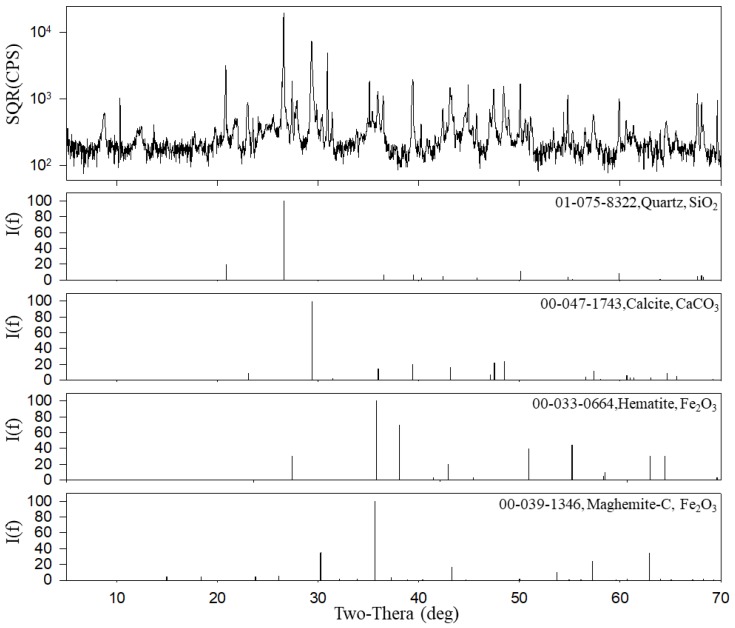
X-ray diffraction (XRD) patterns of PM in the subway tunnel at the S station.

Figure 7 shows the mass concentrations of carbonaceous compounds in PM. The total carbon (TC) contents of PM_10_ and PM_2.5_ were 35.8 ± 7.0 (16.7%) and 18.2 ± 2.6 µg/m^3^ (23.2%), respectively. Meanwhile, TC minus CC represented 16.1% of PM_10_ and 22.3% of PM_2.5_ (Table 3). These concentrations were higher than those of PM in outdoor air. The OC and EC concentrations of PM_10_ were 23.4 ± 3.7 and 11.1 ± 3.8 µg/m^3^, and those of PM_2.5_ were 13.8 ± 2.1 and 3.8 ± 0.8 µg/m^3^, respectively. CC comprised 1.3 ± 0.6 µg/m^3^ of PM_10_ and 0.7 ± 0.3 µg/m^3^ of PM_2.5_. However, it should be noted that the TOT method overestimates the OC content due to the mineral oxides (e.g., Fe_2_O_3_ and SiO_2_) present in the PM in subway tunnels [41]. Carbonate estimated using the ion balance comprised 3.1% of PM_10_ and 2.9% of PM_2.5_ [29,32]. In addition, CaCO_3_ and SiO_2_ were detected by XRD (Figure 8). The carbonaceous components of PM in subway tunnels originate from diesel PM emitted by the diesel motor car operated to maintain the tunnel [16,20], as well as organic material transported from the outdoor air through the ventilation system [16,20], and the pantograph carbon strip–catenary wire interface [4,42].

#### 3.2.2. Ionic Compounds

**Figure 9:**
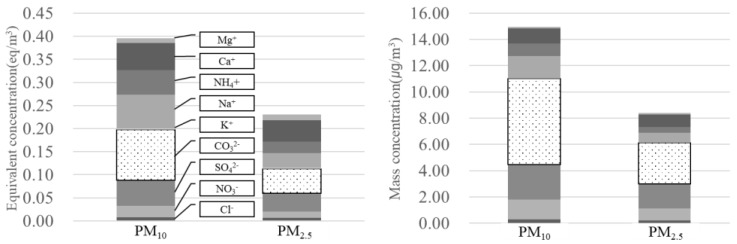
Normal and mass concentrations of ionic components in PM at the M station (*n* = 13).

The mean mass concentrations of ionic compounds in PM_10_ and PM_2.5_ were 11.0 ± 5.1 and 6.7 ± 3.4 µg/m^3^, respectively (Figure 9). The mean normal concentrations of anions (excluding CO_3_^2−^) and cations in PM_10_ were 0.09 ± 0.05 and 0.20 ± 0.09 eq/m^3^, respectively, whereas those in PM_2.5_ were 0.05 ± 0.04 and 0.12 ± 0.06 eq/m^3^, respectively. These results indicated that PM in the subway tunnels contained higher cation than anion (excluding CO_3_^2−^) contents. In this study, CO_3_^2−^ analysis was not conducted, and anions were lost from the filters because of the high temperature and humidity during sampling [45]. For this reason, we estimated the CO_3_^2−^ content based on the cation and anion equivalence [34].

The CO_3_^2−^ concentrations of PM_10_ and PM_2.5_ were 6.6 ± 3.2 and 4.5 ± 1.5 µg/m^3^, respectively. The CO_3_^2−^, SO_4_^2−^, Na^+^, and NO_3_^−^ concentrations in the Seoul subway system during the summer of 2010 were reported previously [29]. Subway tunnels are made of concrete, the debris of which accumulates on the tunnel floor.

#### 3.2.3. Inorganic Compounds

Inorganic compounds represented 47.0% of PM_10_ and 38.2% of PM_2.5_. Fe (principally iron oxides) comprised 40.3% of PM_10_ and 33.6% of PM_2.5_, which was higher than previously reported (36.1% of PM_10_) [16].

**Figure 10:**
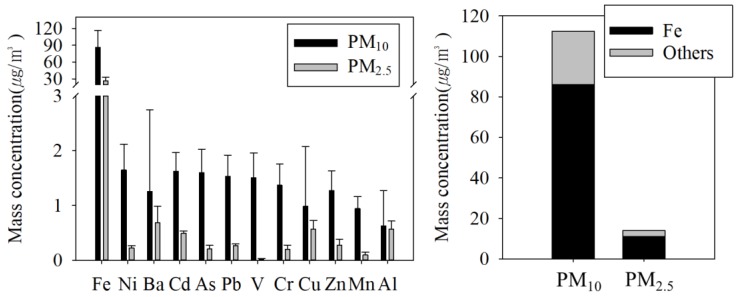
Mass concentrations of inorganic components in PM at the M station.

The mass concentrations of Fe in PM_10_ and PM_2.5_ were 86.1 ± 30.5 and 26.3 ± 6.5 µg/m^3^, respectively, whereas the mass concentrations of other inorganic compounds (Ni, Mn, Ba, Pd, V, Cr, Cu, Zn, and Al ranged from 0.63 to 1.65 µg/m^3^ in PM_10_ and 0.027 to 0.684 µg/m^3^ in PM_2.5_ (Figure 10). Indeed, nano-sized PM were reported to be emitted from the wheel–rail–brake interface [2,5,46].

In addition to iron oxides in PM in the subway tunnel, we detected two iron oxides as hematite (*α*-Fe_2_O_3_) and maghemite-C (*γ*-Fe_2_O_3_) using XRD (Figure 8). Fe_2_O_3_ represented 57.6% of the PM_10_ and 48.0% of the PM_2.5_ in the subway tunnel. Fe in subway tunnels is present as iron oxides and mineral elements; e.g., FeO_x_/SiO_2_ [23]. The estimated mass of Fe_2_O_3_ was 123.1 ± 37.7 µg/m^3^ in PM_10_ and 37.7 ± 9.3 µg/m^3^ in PM_2.5_ (Table 3). In the case of European subway systems, Fe_2_O_3_ is the most abundant element on platforms without PSDs, accounting for 30–66% of the total PM_2.5_, followed by carbonaceous components (18–37%) [32,47]. Fe reportedly also predominates the subway systems of other cities [43,48,49,50].

Meanwhile, Ni, Mn, Ba, Pd, V, Cr, Cu, Zn, and Al together comprised 5.2% of PM_10_ and 3.2% of PM_2.5_. Fe, Ba, and Mn are used in the wheel–rail–brake system, and Ni, V, and Cr are included in lubricant and are generated by oil combustion [28]. The Fe/Mn ratio in PM_10_ was 99.7, which was similar to previous reports regarding rail, wheel, and electric sliding collectors (Fe:Mn ratio, 92–121) [16,48,51,52,53]. Cu, the major component of catenary wire, comprised 0.98 ± 1.09 µg/m^3^ of PM_10_ and 0.53 ± 0.97 µg/m^3^ of PM_2.5_. The carbon strip–catenary wire interface undergoes wear, and a short circuit between the wire and the strip generates an arc discharge [54,55]; according to a previous study [16], electrical cable wear is estimated to contribute about 8.1% of PM_10_.

### 3.3. Morphology and Energy-Dispersive Spectroscopy (EDX)

**Figure 11:**
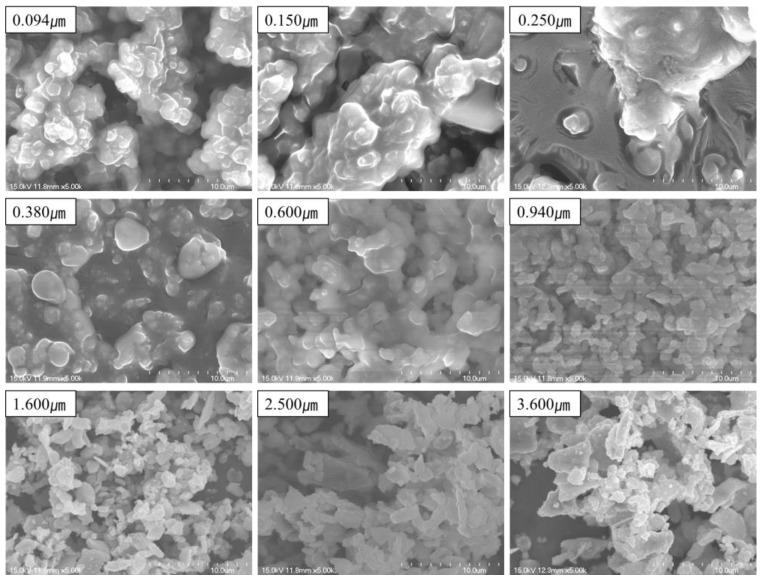
Morphology of PM in subway tunnels according to size at the M station.

We analyzed the morphology of PM in the subway tunnel (Figure 11). PM of 1-µm diameter is considered to be abrasive PM generated by mechanical wear at the wheel–rail–brake interface [24,56]. The majority of dust particles with diameters of below 1 µm PM displayed irregular spheres.

**Figure 12:**
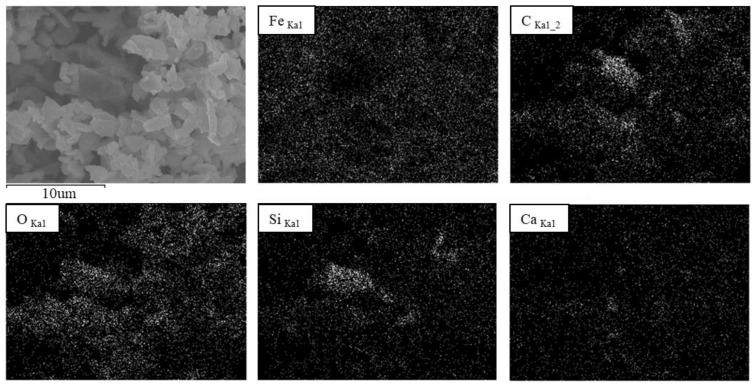
Elemental map of PM_2.5_ at the M station.

An SEM/EDX elemental map of PM_2.5_ is shown in Figure 12. Fe accounted for 44.14 wt.%, followed by C (28.07 wt.%) and O (20.43 wt.%). The Fe component was distributed evenly in the elemental map, suggesting that nano-sized Fe PM generated by friction was condensed with or attached to other PM [18].

Through EDX analysis, Fe was detected in PM ≥ 94 nm (Table 4). Studies showed that 100–200-nm nanoparticles are generated from the wheel–rail–brake interface [2,18]. The ratio of Fe increased with increasing particle size, reaching 47.2% for 1.6-µm-diameter PM. Ca and Si were detected in 380-nm PM. Meanwhile, S was detected in PM ≥ 30 nm, with the highest proportion in 250-nm PM. S entered the subway tunnel from outside [29]. Cu was detected in PM between 94 and 150 nm. To supply electricity, a catenary wire is attached to the pantograph; however, short circuits generate an arc discharge, resulting in the emission of Cu nanoparticles. Cu was also present as copper oxides, e.g., CuO. Notably, CuO nanoparticles have greater toxicity than CuO microparticles [57]. Crustal PM (Ca and Si) ≥ 380 nm could have been generated due to the deterioration of subway facilities and the inflow of soil dust from outdoors. Ba (which is present in brake components) PM of 0.940–2.500 µm was also detected.

According to prior studies [2,18], nanoparticles are generated at sites of wheel–rail contact that surpass a given temperature threshold as the train brakes. It is assumed that nanoparticles were emitted at the wheel–rail contact rather than the brake pad–disc interface due to the difference in Ba and Fe particle size.

According to the XRD patterns (Figure 8), the PM in subway tunnels comprised calcium carbonate (CaCO_3_; JCPDS card number, 00-047-1743), quartz (SiO_2_; 01-075-8322), hematite (*α*-Fe_2_O_3_; 00-033-0664), and maghemite-C (*γ*-Fe_2_O_3_; 00-039-1346). Calcium carbonate is a major component of cement, and quartz is the second most abundant mineral in continental crust. Upon exposure to air, Fe is oxidized into iron oxide or iron hydroxide; the latter is reportedly present on the surface of rails in the Tokyo metro [58]. Previous studies reported the presence of calcium carbonate (CaCO_3_), quartz (SiO_2_), hematite (*α*-Fe_2_O_3_), maghemite (*γ*,*ε*-Fe_2_O_3_), magnetite (Fe_3_O_4_), Fe, goethite (*α*-FeOOH), and akaganeite (*β*-FeOOH) in PM [24,49,58,59,60]. However, in this study, we did not detect Fe, goethite, akaganeite, or magnetite using XRD spectroscopy.

**Figure 13:**
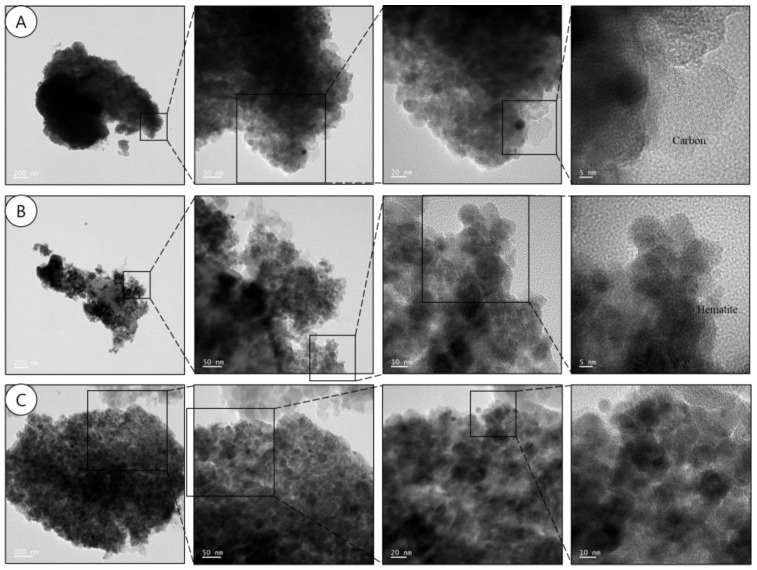
Morphology of PM in subway tunnels according to size at the S station.

TEM images of PM in the subway tunnels showed flakes of iron oxide nanocrystals (Figure 13). According to previous reports and the XRD patterns, iron oxide PM contains various chemical species, e.g., hematite (*α*-Fe_2_O_3_), magnetite (Fe_3_O_4_), and goethite (*α*-FeOOH) [23,58]. The PM showed mottling of clusters of rounded ferruginous nanocrystals a few nanometers in width, along with highly crystalline magnetite, hematite, and C nanocrystals. According to Moreno [23], Fe nanoparticles are generated mechanically by frictional wear, especially through the sliding of two metallic surfaces, followed by oxidation, while others suggested their generation via condensation of Fe vapor [18,24,30,61,62]. Magnetite crystals have octahedral and rhombic dodecahedral morphologies, whereas maghemite nanoparticles show an elongated cuboctahedral morphology [59]. Table 5 shows the chemical compositions of PM in Figure 13. The PM contained Fe and O (Table 5). The elemental ratios of particles A and B were similar, while particle C presented comprised 71.32% Fe and 16.33% O. C was excluded due to the use of a TEM grid composed of Cu and C. However, concentric C nanocrystals were detected in particle A (Figure 13).

## 4. Conclusions

In summary, Fe was the most abundant element in the subway tunnels, accounting for higher proportions of PM; Fe was detected in PM with diameters >94 nm, and was observed as mottling of clusters in rounded ferruginous nanocrystals with a few nanometers. Fe was present mostly as iron oxides, which were emitted from the wheel–rail–brake interface. Copper particles were 96–150 nm in diameter and were likely emitted via catenary wire arc discharges. The carbonaceous components of PM in subway tunnels originate from diesel PM emitted by the diesel motor car operated to maintain the tunnel. Organic material was transported from the outdoor air through the ventilation system and also originated from the pantograph carbon strip–catenary wire interface. Ca and Si in the subway tunnel, present as calcium carbonate (CaCO_3_) and quartz (SiO_2_), were detected in PM with diameters >380 nm. Calcium carbonate (CaCO_3_) is a major component of cement, and quartz is the second most abundant mineral in continental crust.

In the near future, our research team will conduct measurements at three locations (outdoors, platform, and tunnel) to identify PM exposure to passengers for each location.

These findings suggest that the majority of nano- and micro-PM in the subway tunnel in Seoul, South Korea is generated due to the operation of the subway. Our results can be used to improve the indoor air quality (IAQ) in subway tunnels and to prevent the generation of PM due to subway operation.

## Figures and Tables

**Table 1:** Measurement devices and sampling periods.

Item	Filter	Particle Diameter	Flow Rate (L/min)	Analysis Instruments	Sampling Period and Site
Low-volume air sampler (PMS-104)	Zefluor	PM_10_ and PM_2.5_	16.7	Ion compound (IC), Metal compound (ICP-AES)	15 May to 9 June 2017 (*n* = 13) M station, Seoul
Mini-volume air sampler (MiniVol TAS)	Quartz	PM_10_ and PM_2.5_	5.0	Organic and Elemental Carbon (Carbon analyzer)	
Electrical low-pressure impactor (ELPI)	Aluminum foil	14 stages (D_50_^1^: 10, 5.3, 3.6, 2.5, 1.6, 0.94, 0.60, 0.38, 0.25, 0.15, 0.094, 0.054, 0.030, and 0.016 µm)	10.0	Morphology and Chemical by size distribution (SEM and TEM/EDX)	15 May to 17 May 2017 M station, Seoul
Low-volume air sampler PMS-104	Zefluor	PM_10_	16.7		18 April 2018 S station, Seoul
Sieve (mesh 18: Φ = 1.00 mm)	Zipper bag	Floor dust under 1 mm	-	Chemical form (XRD)	Three times within 15 May to 9 June 2017 S station, Seoul

^1^ D-Values (D_50_) are the intercepts for 50% of the cumulative mass. PM—particulate matter; ICP-AES—inductively coupled plasma atomic emission spectrometry; EDX—energy-dispersive X-ray spectroscopy; XRD—X-ray diffraction; SEM—scanning electron microscopy; TEM—transmission electron microscopy.

**Table 2:** NIOSH Method 5040 parameters.

Program Activity	Carbon	Carrier Gas	Ramp Time (s)	Program Temperature (°C)
Oven purge	-	He	10	Ambient
1st ramp	OC1		60	315
2nd ramp	OC2		60	475
3rd ramp	OC3		60	615
4th ramp	OC4 and CC		90	870
Cooling for EC	CC		30	0
Stabilize temp	PC and EC	He/O_2_	45	550
He/O_2_ 1st ramp	PC and EC		45	625
2nd ramp	EC		45	700
3rd ramp			45	775
4th ramp			45	850
5th ramp			120	910
External standard, calibration and cool down	-	Calibration gas and He/O_2_	120	0

OC—organic carbon; CC—carbonate carbon; PC—pyrolytic carbon; EC—elemental carbon.

**Table 3:** Physical and chemical characteristics of PM at the M station (*n* = 13).

	PM_10_				PM_2.5_				
	This Study		In Seoul [16]	In Mexico City [43]	This Study		In Seoul [29]	In Barcelona [44]	In Mexico City [43]
	Mass (g/m^3^)	Ratio (%)	Mass (g/m^3^)		Mass (g/m^3^)	Ratio (%)	Mass (g/m^3^)		
Total	213.7 ± 50.4	100	200.75	89.55	78.4 ± 8.8	100	55.1	20.7–93.2	48.34
TC *	34.5 ± 6.8	16.1	-	-	17.5 ± 2.6	22.3	-	3.2–17.1	-
Anion **	11.0 ± 5.1	5.2	18.16 ^+^	-	6.7 ± 3.4	8.6	6.4 **	-	-
Cation	4.0 ± 1.8	1.9	9.82 ^++^	-	2.5 ± 1.3	3.2	3	-	-
Inorganic ***	11.1 ± 1.5	5.2	8.68 ^+++^	-	2.9 ± 0.6	3.7	-	-	-
Fe	86.1 ± 30.5	40.3	72.51	5.57	26.3 ± 6.5	33.6	-	-	3.1
Fe2O3 ****	123.1 ± 37.7	57.6	-	-	37.7 ± 9.3	48	-	6.9–52.4	-
Unknown	30.0 ± 22.1	14	-	-	11.1 ± 9.7	14.1	-	-	-

* Excluding carbonate C; ** including carbonate C; *** excluding Fe; **** Fe as Fe_2_O_3_; ^+^ Cl^−^, NO_3_^−^, and SO_4_^2−^; ^++^ Na^+^, K^+^, Mg^2+^, and Ca^2+^; ^+++^ Al, Ba, Cr, Cu, Fe, Mn, Ni, Pb, Si, Ti, and Zn. TC—total carbon.

**Table 4:** Chemical composition of PM according to size at the M station (unit, %).

Particle Size (µm)	C _K_	O _K_	S _K_	Fe _K_	Cu _L_	Ca _K_	Si _K_	Ba _L_	Br _L_	K _K_	Mo _L_	Mg _K_
0.016	91.58	7.21									1.21	
0.030	84.81	12.25	2.94									
0.054	95.65	4.35										
0.094	80.95	11.49	3.39	1.73	1.89					0.56		
0.150	83.17	8.62	1.48	1.45	1.87				3.42			
0.250	70.67	8.26	8.86	12.21								
0.380	64.03	17.95	1.55	14.80		0.7	0.96					
0.600	60.89	14.31	0.87	20.54		1.32	1.27		0.80			
0.940	40.37	18.65	0.78	34.19		2.06	1.83	2.11				
1.600	21.69	22.97	0.79	47.20		2.38	3.09	1.87				
2.500	28.07	20.43	0.60	44.14		1.95	2.73	1.64				0.44
3.600	38.62	18.28	0.76	37.96		1.63	2.74					

_K_, _L_ is principal quantum number of elemental.

**Table 5:** Chemical compositions * of three samples of PM in Figure 13 (unit, %).

Element	A	B	C
Iron (Fe)	43.98	43.94	71.32
Oxygen (O)	40.40	40.58	16.33
Silicon (Si)	6.59	6.59	3.71
Magnesium (Mg)	0.34	0.34	1.42
Aluminum (Al)	1.18	1.17	1.76
Calcium (Ca)	1.77	1.76	
Sodium (Na)	2.80	2.79	
Phosphorus (P)	0.68	0.68	
Sulfur (S)	0.47	0.47	
Chlorine (Cl)	0.54	0.54	
Potassium (K)	1.14	1.14	
Titanium (Ti)	0.12		
Zirconium (Zr)			
Sum	100.00	100.00	100.00

* Cu and C were excluded due to the use of a Cu–C TEM grid.
